# A teacher-led classroom intervention in an area of Mexico experiencing community violence: A controlled mixed-method feasibility study

**DOI:** 10.1371/journal.pone.0323562

**Published:** 2025-06-09

**Authors:** Suzanne M. Connolly, Leonor Zarazúa Menchaca, Héctor Figueroa Palafox, Heather L. Adams, John Freedom

**Affiliations:** 1 Research Committee, Association of Comprehensive Energy Psychology, Bryn Mawr, Pennsylvania, United States of America; 2 Department of Community Projects, San Andrés Pesc ador Asociación, Ciudad de México, México; 3 Department of Poverty Analysis, Guidelines for the Measurement of Poverty and Social Development, National Council for the Evaluation of Social Development, Ciudad de México, México; 4 Research Studio LLC, Fort Wayne, Indiana, United States of America; Health Researcher, SPAIN

## Abstract

**Objectives:**

We aimed to explore the effectiveness of a daily ten-minute teacher-led group thought field therapy stress-reduction intervention on middle-school adolescents residing in an area experiencing high levels of interpersonal and community violence. We hypothesized that it would lead to a reduction of trauma symptoms and improve grades in reading and math.

**Methods:**

In this double-masked feasibility study, adolescents in one school received a daily teacher-led thought field therapy intervention, and children in a different school served as an active waitlist group and received the same amount of time in a daily unguided drawing activity. The schools were geographically distant to prevent cross-contamination.

**Results:**

Due to differences between groups in PTSD and academic performance prior to intervention, differences within each school’s scores over time were calculated and compared to each other for indirect assessment of effect. PTSD scores at the treatment school showed no lasting change at five months, while the control school showed moderate improvement. Adolescents in the treatment group demonstrated large improvements in both reading and math. Adolescents in the control group demonstrated moderate decrease in math, and no change in reading.

**Conclusions:**

Preliminary evidence gained in this study suggests that a teacher-led ten-minute group thought field therapy exercise might assist adolescents’ learning in reading and math. However, in an area experiencing ongoing interpersonal and community violence, a more multicomponent approach with longer periods of intervention, caretaker involvement and individual therapy for those who might benefit from it, may be needed.

Study registration number: ISRCTN10548974.

## Introduction

Adverse childhood experiences (ACEs) shorten life span, increase risky behavior, and increase the likelihood of disease [1]. ACEs include personal losses, neglect, undergoing medical procedures, and experiencing accidents resulting in personal injury and exposure to natural disasters. They also include experiencing interpersonal violence, such as sexual, emotional, or physical abuse, exposure to war, and community violence such as police violence, and gang activity. In addition to health risks resulting from exposure to ACES, dose–response effects between the number of ACEs and risk of decreased school attendance, and lower performance in reading and math [2], and school suspensions [3] have been documented. This study attempts to study a simple teacher-led intervention to reduce symptoms of trauma and increase academic performance as measured by reading and math scores in middle school children in an area of Mexico experiencing high rates of interpersonal violence.

### Literature review

Interpersonal violence is one of the largest contributors to death and disability for adolescents and young adults worldwide. In 2019, interpersonal violence ranked 26th in the list of the leading contributors to disability-adjusted life years (DALYs) in the general population. However, in the same year, interpersonal violence ranked 5th in terms of DALYs for adolescents and young adults aged 10–24 [4]. Interpersonal violence is one of the leading causes of DALYs for adolescents and young adults worldwide; however, it is reflected unevenly geographically and in time. In 2019 interpersonal violence was the 7th largest cause of death and the 4th largest contributor to DALY’s in the general Mexican population [5].

One study of family violence in Monterrey, Mexico, found high incidences of both interpersonal and community violence [6]. According to the authors, the most frequently reported traumatic experiences reported were: “seeing someone being beat up, shot at, or killed in town and seeing a dead body in town (not including funerals)”.

Community violence has been found to have an even stronger impact on academic achievement than interpersonal violence, and that the degree of community violence correlates with lower academic achievement [7]. In a study of sixth-grade middle-school children, witnessing violence was found to have an even greater impact on academic performance than being the victim of violence [8].

More specifically, a study conducted in Mexico examined the effects of drug-trade-related homicides and non-drug-related homicides on academic outcomes in primary and secondary schools. These authors found a significant geographic correlation between the number of homicides in a community and standardized academic test scores. They found that the effect size was larger in secondary schools, and that effect sizes grew larger in relation to the crime’s proximity to schools [9].

Given the prevalence of mental health conditions in children and adolescents globally, it has long been suggested that greater attention be given to children and adolescents’ mental health issues overall, especially in low and middle-income countries (LMICs) where 90% of the world’s children and adolescents live, and where children tend to have the greatest exposure to violence [10].

However, the number one obstacle to mental health care in LMICs is the scarcity of professional mental health workers [11]. As Mexico has rapidly evolved to becoming an upper middle-income country with the 15^th^ largest economy in the world [12], the gap between the prevalence of health treatment needs, in general, and access to all health treatment including mental health treatment is closing. Due in part to income disparities, however, the gap remains significant [5].

### One proposed solution

One proposed solution to help minimize mental health treatment shortages in LMICs is the utilization of professionally trained lay counselors, drawn from the communities they live and work in, building on and strengthening community capacity [13,14]. Although participants in studies utilizing mental health professionals tend to perform better than those utilizing lay counselors [10], due to the scarcity of professional resources in LMICs, the training of lay counselors needs to be prioritized.

A 2021 meta-analysis of randomized controlled trials (RCTs) published by the World Health Organization found that studies of lay-counselor mental health interventions in LMICs can and often do achieve medium and high effect sizes [13]. The authors found that of the 20 lay counselor interventions analyzed, six demonstrated medium effect sizes and five demonstrated large effect sizes. Three of the included studies in the meta-analysis utilized thought field therapy (TFT) procedure where the relaxation response is promoted by tapping on specific acupoints on the face and hands. Of the three included TFT studies, one demonstrated a medium effect size and two demonstrated large effect sizes. We chose TFT as our evidence-based intervention based on personal experiences using TFT for the treatment of trauma and other mental health issues in LMICs [15–17], its ease of training lay counselors, and its relative effectiveness [12,15–17].

A review of 36 studies conducted in LMICs looked at studies of children and adolescents after human-made or natural disasters [18]. It included both RCTs and pre-post trials including one TFT intervention study with former street children, delivered by a combination of mental health workers and lay counselors. They found the TFT study to have the highest effect size, when compared to other pre-post trauma interventions included.

We chose to employ a teacher-led classroom-based TFT group intervention due to scarce mental health resources, ease of delivery, and scalability. We saw scalability as an important part of the study. Our aim was to create a model that could be replicated first on a local scale in this area of Mexico, and eventually on a national and global scale.

In a review of 42 papers on sustainability and scalability of social innovation [19], common findings were the importance of flexibility from area to area, local autonomy, cost-effectiveness, and building strong networks. In this study, we worked with a limited budget and collaborated with local community leaders with strong established networks.

We hypothesized that a 10-minute per school-day teacher-led exercise in TFT would significantly reduce trauma symptoms in middle-school students in the TFT group when compared to reduction of trauma symptoms in the 10-minutes per school-day art activity intervention in the active waitlist group. We also hypothesized that grades in reading and math would significantly improve in the treatment group compared to grades in reading and math in the art-activity active waitlist group [20]. Only our second hypothesis was supported.

## Materials and methods

### Participants and demographics

The principals of two middle schools in an area geographically separated but with similar demographics accepted a verbal and written invitation from their school superintendent to participate in the study and then signed an invitation to participate in one of two studies. [see Appendices A & B in S1 File]. Inclusion criteria was being a middle school (classes 6 and 7) located in the municipality of Ziracuaretiro with a principal agreeing to participate and at least one teacher in the school willing to participate. There are 5 middle-schools in the school district of Ziracuaretiro and the superintendent decided to choose the two schools based on population need such as trauma, kidnapping, and disappearance of family members; thus, the study was not cluster-randomized as originally planned. The individual classroom response was suboptimal as only three teachers agreed to take on an extra duty during their workday. The two schools were not randomized as planned as one teacher agreed to participate only in the art activity group.

Participants were 99 boys and girls aged 11–14, who attended middle schools in the municipality of Ziracuaretiro, in the state of Michoacán de Ocampo. The study was approved prior to the study’s beginning by the Ethics Committee of the Universidad Intercultural Indigena de Michoacan (UIIM) [see Appendix C in S1 File].

The adolescents and their caretakers signed informed consent forms [see Appendices D, E, F, & G in S1 File], and were given no clues in their invitations or on their consent forms as to which school group was the treatment group and which school group was the active waitlist group. The invitations explained that two different approaches to creating a calm learning environment will be studied and compared; one approach at one school and the other approach at the other school, and that we were testing possible ways to reduce the effects of trauma and enhance the learning ability of middle-school adolescents. The invitations and consents also explained that at the study’s finish, if the improvement in one school classrooms significantly surpassed the improvement in the other school’s classrooms, all teachers who wish to participate will be given the opportunity to be trained to conduct the more effective intervention in their classrooms for the following semesters. Teachers were provided adverse experience forms to record any adverse experiences as a result of participating in the trial [see Appendix H in S1 File].

As the areas of the consenting schools neighbor the municipality of Uruapan, second in the state of Michoacán with the most recorded criminal offenses [21], the adolescents and their caretakers live in circumstances that expose them to both interpersonal and community violence.

### Design

This study was not cluster-randomized as planned as one teacher agreed to participate only in the art activity group. Our study follows the Transparent Reporting of Evaluations with Nonrandomized Designs (TREND guidelines). Originally designed as a collaborative quantitative study, due to emerging factors the study developed as a mixed methods study [22]. The two originally agreed upon main authors decided, due to their busy teaching schedule, that they could not participate in the quantitative study, but wanted to follow-up with a qualitative study, which, as professors of anthropology, they were more accustomed to working with a mixed method, explanatory sequential design was mutually agreed on. The first phase of the design was the collection and analysis of the quantitative data followed by the qualitative authors’ gathering their data via structured interviews and qualitatively analyzing their data. The qualitative study was published prior to the quantitative study in a Mexican educational journal in the Spanish language [23].

The treatment group intervention consisted of a daily, ten-minute group classroom TFT (acupoint tapping) intervention guided by the teacher each school day. The active waitlist group intervention consisted of a ten-minute morning teacher initiated unguided group art activity consisting of colored marker pen artwork. The teachers in both classrooms spent an equal amount of time with the respective interventions and no additional class time was added to their schedules.

**Fig 1:**
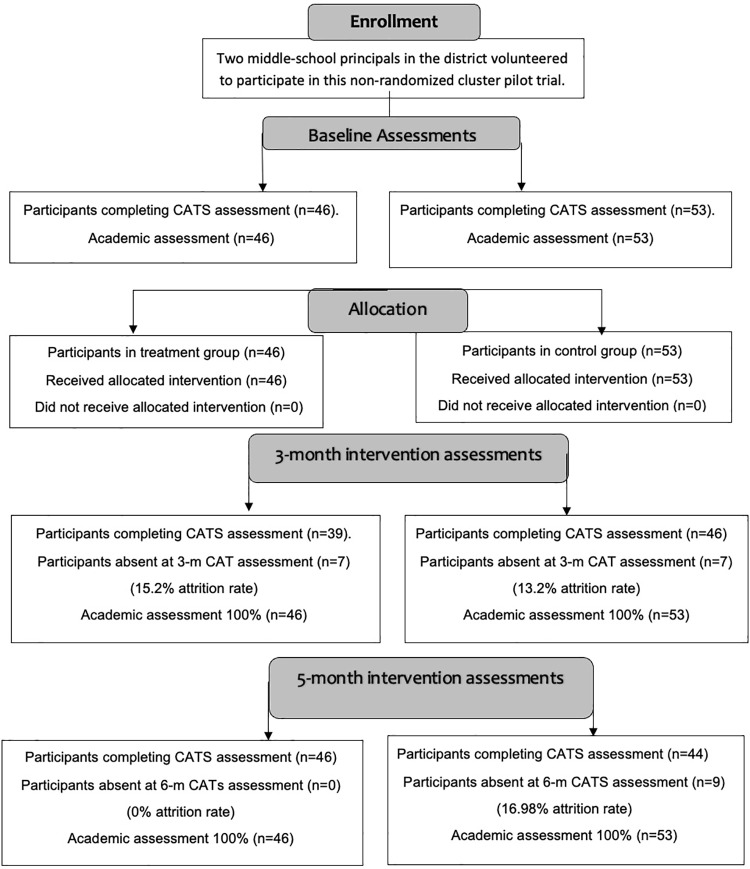
Non-randomized trial assessments.

The pre-assessments took place during the fourth week of the school semester and the interventions began the following week. The first post-assessment took place three months later, and the second post-assessment at five months during the last week of the fall semester. Teachers recorded students’ grades at their usual times, at the end of the first quarter, second quarter, and third quarter (Fig 1). Originally designed as a six-month study, local circumstances delayed the initial assessment and caused the semester to end earlier than initially planned.

The adolescents and their caretakers were assessed by UIIM graduate students using the Spanish version of the Child and Adolescent Trauma Screen (CATS): Youth Report 7–17 and CATS Caretakers Report 7–17 [24,25]. [see Appendices I & J in S1 File]. Due to hours of parents and caretakers working in *maquiladoras* and agriculture, the Caretaker’s Report was included as a way to involve parents and caretakers who could participate, but it was not part of the analysis. The CATS tools track ongoing exposure to adverse childhood experiences including interpersonal and community violence and tracks current trauma symptoms as experienced in the prior month. It has been translated and validated in the Spanish language [26].

The second author, who served as the study coordinator occasionally videotaped teacher-led sessions in both schools to assess for treatment fidelity, videotaping the teachers and blocking out any faces of any adolescent participants before sharing with the authors.

In the sequential qualitative study, published separately in Spanish, semi-structured interviews were conducted by the qualitative study authors with 44 persons who participated in the treatment group: 28 middle school students, three teachers, ten mothers and two grandmothers, and the principal of the school. The interviews were conducted privately, in an isolated classroom at the treatment school. The aim of these local investigators was to qualitatively explore the efficacy of the TFT stress-reduction intervention in reducing trauma symptoms and improving reading and math scores [23].

### Treatments and teacher training

TFT is a self-administered acupoint tapping procedure. The person receiving the procedure is instructed to tap with their fingers on specific acupoints, one at a time in a specific order. Five points are located on the face, one on the side of the body, one on the upper chest, two on the fingers, and two on the hand. The teachers received the basic two-day training in TFT, led by the first author and translated into Spanish by the second author. The standardized TFT training manual translated into Spanish [see Appendix K in S1 File]. was used for the training. The teachers were given a simplified script [see Appendix L in S1 File] that was appropriate for group intervention to use in the trial. The teachers were instructed to begin the group exercise by informing the adolescent participants to think about anything that might be bothering them and getting in the way of their learning or their ability to be happy, while they tapped on themselves.

The adolescents in the active waitlist group received 10 minutes of a free-form art activity daily. The classroom was supplied with art paper and colored marking pens. The teacher and school staff were instructed in a short training by a trained art therapist who cautioned the teacher to not consider this art “therapy”, but a form of free expression; and also, not to comment positively or negatively on the drawings.

### Assessments and assessor training

Six trained graduate students from the UIIM administered the CATS assessments to the adolescents and their caretakers. The graduate students were trained to administer the CATS in a four-hour training [see Appendix M in S1 File] held at the UIIM, led by two professors and the second author. During the training, the graduate students were not exposed to the questions contained in the assessments in order to keep the assessment masked. The six graduate students received university credit for their participation but no financial compensation. The graduate students were supervised professionally throughout the assessments by their two professors, with on the ground practical assistance from the second author. The graduate student assessors were unaware of which group was considered the treatment group, and which group was considered the active waitlist group.

The statistician programmed iPad tablets that allowed the adolescents and caretakers to read and answer one interview question at a time. The question then disappeared into a secure database and directed the adolescent or caretaker to the next question and so on to the end of the survey. The graduate student assessors were present to provide instructions, support, to answer questions the participants might have, and to listen should a participant want to discuss a question or answer further. The University professors involved in the study design felt that this method would increase the likelihood of more accurate answers, while at the same time the interviewer remained there during the assessment for students who wanted to discuss their situation further. There were no traces of the answers on the electronic devices. The assessment had only a number with no name attached, and the statistician had no access to the name/number key. Only the second author had access to the name-number key which was kept in a secure locked location at the UIIM.

The graduate students assessed the students and their caretakers at their schools in secluded areas with only the adolescent or caretaker (never both at the same time) present at appointed times. The students were assured of confidentiality by the graduate-student-assessor who asked the students to answer each question carefully. The graduate-student-assessors did not see the adolescents’ or caretakers’ questions or answers. The adolescent or caretaker was not asked to talk about any of the questions, but if they did, the graduate-student-assessor was instructed to listen carefully and be empathetic and supportive.

### Assessing data set quality and comparability across sites

The treatment condition began with 46 students (see Table 1 for all sample sizes) completing the CATS. This dropped to a low of 39 at the three-month period, producing a 15.2% attrition. The control condition began with 53 students completing the CATS. This dropped to a low of 44 at the six-month period, producing a 16.98% attrition. The academic assessments had 0% attrition in both conditions.

**Table 1:** Assessment of Normality of all data set with Shapiro-Wilks (*W*).

Treatment Data Sets	*n*	*W*	*P*-value^a^	Control Data Sets	n	*W*	*P*-value^a^
Trauma Experiences				Trauma Experiences			
Baseline	46	.920	<.001	Baseline	53	.937	.01
3 months	39	.958	.16	3 months	46	.920	<.001
5 months	46	.952	.01	5 months	44	.911	<.001
PTSD Total Score				PTSD Total Score			
Baseline	46	.879	<.001	Baseline	53	.966	.14
3 months	39	.888	<.001	3 months	46	.917	.00
5 months	46	.901	<.001	5 months	44	.910	.00
Subscales				Subscales			
Re-Experiencing				Re-Experiencing			
Baseline	46	.718	<.001	Baseline	53	.683	<.001
3 months	39	.578	<.001	3 months	46	.650	<.001
5 months	46	.634	<.001	5 months	44	.609	<.001
Avoidance				Avoidance			
Baseline	46	.647	<.001	Baseline	53	.643	<.001
3 months	39	.446	<.001	3 months	46	.664	<.001
5 months	46	.559	<.001	5 months	44	.451	<.001
Negative Mood/Cognition				Negative Mood/Cognition			
Baseline	46	.861	<.001	Baseline	53	.867	<.001
3 months	39	.861	<.001	3 months	46	.896	<.001
5 months	46	.830	<.001	5 months	44	.824	<.001
Arousal				Arousal			
Baseline	46	.809	<.001	Baseline	53	.898	<.001
3 months	39	.791	<.001	3 months	46	.855	<.001
5 months	46	.829	<.001	5 months	44	.670	<.001
Functional Impairment				Functional Impairment			
Baseline	46	.690	<.001	Baseline	53	.849	<.001
3 months	39	.804	<.001	3 months	46	.888	<.001
5 months	46	.787	<.001	5 months	44	.796	<.001
Academics				Academics			
Mathematics				Mathematics			
Baseline	50	.870	. < 001	Baseline	50	.909	.001
3 months	50	.793	<.001	3 months	50	.922	<.001
5 months	50	.895	.001	5 months	50	.910	.001
Reading				Reading			
Baseline	50	.796	<.001	Baseline	50	.895	<.001
3 months	50	.885	<.001	3 months	50	.914	.001
5 months	50	.878	<.001	5 months	50	.861	<.001

^a^threshold for significance p < 0.05.

Student attrition produced multivariate, nonmonotone missing data of a random nature. Despite high rates of absenteeism in Mexican schools [26], in both conditions, attrition rates for the CATS were less than 20%, indicating acceptable external validity. Comparability of attrition rates between treatment and control conditions indicates no threats to internal validity. Due to the correspondence between attrition rates and missing data, the percentages of missing data exceeds the 10% cut off for employing imputation of means without undue risk of introducing bias to the data set [27]. Furthermore, no method of pattern substitution of maximum likelihood estimation is currently recommended for imputing missing data for the CATS. A Shapiro-Wilk test of normality [28] assessed assumption of normality, due to sample size < 50.

Mann-Whitney analysis [28] of differences between the schools’ pre-intervention scores on all measurements assessed comparability of sites. Because no parametric assumptions are required for nonparametric tests, such as the Mann-Whitney, a confidence interval of the median differences between two groups can be unreliable, and misleading, and thus rarely reported. For example, the SPSS computation of the differences in the Function sub-scale scores between the treatment group’s baseline and corresponding 5-month assessment produces a median of −5, with a lower limit value of −1, and upper limit value of 0. The location of the measure of central tendency far outside the 95% confidence interval values violates the purpose of a confidence interval to provide a range of values in which the true parameter is plausibility contained. Due to the unreliability of the confidence interval with nonparametric tests, this calculation was excluded from the analysis plan.

### Testing the hypotheses

Differences in pre-intervention scores on some measures (see Table 2) altered plans to directly compare trauma symptomatology and academic performance between the two schools. Instead, changes within each school were assessed, and statistically significant changes, effect sizes and directions of change were then compared between the two schools to provide an indirect assessment of possible treatment impact. The Wilcoxon Signed Ranks test [28] was determined as the optimal analysis approach for this data. This required adjusting the hypotheses of the study from directional 1-tail to nondirectional, as the Wilcoxon Signed Ranks test is inherently a 2-tail test. Benjamini-Hochberg adjusted P-value was used to correct for multiple comparisons [29]. Briefly, the P-values for each pair of comparisons are placed in rank order. The larger P-value is compared to the original statistical significance criteria. The smaller P-value is compared to the adjusted P-value, which is calculated by dividing the original statistical significance value by the number of comparisons made. For example, for the Function Subscale in Table 1, the baseline-5 months P-value of .04 is greater than the baseline-3 months P-value of .002. Thus, .04 is compared to the original significance level of .05, while .002 is compared to .025. The Calculation of *r* was the best option for assessing effect size for nonparametric tests [29]. Interpretation of *r* corresponds with Pearson’s correlation coefficient [30]. Similar to the Mann-Whitney, because no parametric assumptions are required for the Wilcoxon Signed Ranks test, associated confidence intervals can be unreliable and thus were excluded from the analysis plan.

**Table 2:** Mann-Whitney assessment of comparability of treatment and control schools prior to initiating intervention.

	Treatment School median (IQR)	Control School median (IQR)	*U*	*P*-value^a^
Trauma Experiences	3 (2)	3 (3)	1139.50	.58
PTSD Scale	6 (6)	8 (5)	1086.5	.35
Re-experiencing subscale	0 (1)	0 (1)	1200	.88
Avoidance subscale	0 (1)	0 (1)	1221	.96
Negative Emotions subscale	1(3)	1(1)	1112	.44
Arousal subscale	1(2)	2 (2)	869	.01
Function subscale	5 (1)	4 (2)	1548	.02
Mathematics	6 (3)	8 (3)	543	<.001
Reading	7 (2)	8 (2)	810.5	.02

^a^threshold for significance p < o 0.05.

## Results

### Assessing data set quality and comparisons across sites

Shapiro-Wilks test of normality revealed that all but two of the data distributions were non-normal (see Table 1), indicating nonparametric tests as most appropriate.

As presented in Table 2, there were no significant differences between the schools on trauma experiences, indicating that school-site would not function as a potential confounding variable.

Looking at trauma symptoms, no significant differences in student’s PTSD scores, or scores on the Re-Experiencing, Avoidance, or Negative Mood/Cognition subscales were detected between the two schools. However, students’ scores on both the Arousal and the Functional Impairment subscales did differ between the treatment and control sites. Students’ academic performance differed between the schools, with students at the treatment school having significantly lower pre-trial scores in both mathematics and reading, when compared to students at the control school.

### Testing of hypotheses

#### Assessment of impact from baseline to three months intervention.

Starting with the trauma symptoms, students at the treatment school showed a moderate decrease in the Avoidance and the Functional Impairment subscales (see Table 3 for analysis values). No statistically significant changes were evident for PTSD score, Re-Experience, Negative Emotions, or Arousal subscales. The control school showed a different pattern, with no statistically significant changes in the PTSD score or any of its five subscales (see Table 4 for analysis values). Turning to academic performance, students at the treatment school showed a large improvement in Mathematics, along with a moderate improvement in Reading. No significant change in reading scores was evident at the control school, while mathematics performance decreased moderately. Analysis detected no statistically significant changes in trauma experiences at either location, removing this potential confounding variable.

**Table 3:** Treatment school, Wilcoxon signed ranks analysis of differences between (a) Baseline to 3 months intervention, and (b) Baseline to 5 months intervention.

	Baseline Median (IQR)	3 Months Median (IQR)	6 Months Median (IQR)	Baseline – 3 Months			Baseline – 5 Months		
				*W* (*p*-value)	*r* effect size^a^	Direction of Change	*W* (*p*-value)	*r* effect size^a^	Direction of Change
Traumatic experiences	3 (2)	4 (3)	3 (2)	488.5 (.45)^b^	–	–	340 (.91)^c^	–	–
PTSD score	6 (6)	7 (3)	7 (4.5)	622.5 (.03)^b^	–	–	364.5 (.62)^c^	–	–
Re-Experience Subscale	0 (1)	0 (·5)	0 (1)	295.5 (.03)^b^	–	–	72 (.06)^c^	–	–
Avoidance Subscale	0 (1)	0 (0)	0 (.5)	211.5 (.02)^b^	.35	decrease	545 (.51)^c^	–	–
Negative Emotions subscale	1 (3)	1 (3)	1 (2.5)	388.5 (.38)^b^	–	–	243.5 (.93)^c^	–	–
Arousal Subscale	1 (2)	1 (2)	1 (2.5)	323.5 (.44)^c^	–	–	105 (.29)^b^	–	–
Function Subscale	5 (1)	4 (2)	4 (2)	498.5 (.002)^b^	.49	decrease	273 (.04)^c^	.30	decrease
Mathematics	6 (3)	7 (3)	8 (2)	133.5 (<.001)^b^	.50	increase	105 (<.001)^c^	.66	increase
Reading	7 (2)	8 (1)	8 (3)	200 (.03)^c^	.32	increase	88 (<.001)^b^	.62	increase

^a^Pearson r effect sizes: (a) ·00 indicates no effect size; (b) ± ·.01 to ± ·29 indicates a small effect size; (c) ± ·30 to ± ·49 indicates moderate effect size; (d) ± ·50 to ± 1 indicates large effect size.

^b^Benjamini-Hochberg adjusted significant level due to multiple comparisons is p < 0.025.

^c^Benjamini-Hochberg adjusted significance level due to multiple comparisons is p < 0.05.

**Table 4:** Control school, Wilcoxon signed ranks analysis of differences between (a) Baseline to 3 months intervention, and (b) Baseline to 5 months intervention.

	Baseline Median (IQR)	3 Months Median (IQR)	6 Months Median (IQR)	Baseline – 3 Months			Baseline – 5 Months		
				W (*P*-value)	*r* effect size^a^	Direction of Change	W (*P*-value)	*r* effect size^a^	Direction of Change
Traumatic Experiences	3 (3)	3.5 (4.75)	3 (2.25)	635.5 (.45)^b^	··	··	715 (.55)^c^	··	··
PTSD score	8 (5)	8 (6.75)	6.5 (4)	762 .52)^c^	··	··	912.5 (.02)^b^	.35	decrease
Re-Experience Subscale	0 (1)	0 (1)	0 (1)	418.5 (.47)^c^	··	··	339 (.07)^b^	··	··
Avoidance Subscale	0 (1)	0 (1)	0 (0)	288.5 (.41)^c^	··		208.5 (.01)^b^	.42	decrease
Negative Emotions subscale	1 (1)	2 (3.75)	1 (3)	457.5 (.50)^c^	··	··	737.5 (.06)^b^	··	··
Arousal Subscale	2 (2)	1 (3)	1 (1)	553.5 (.20)^c^	··	··	696 (.01)^b^	.41	decrease
Function Subscale	4 (2)	4 (1)	4 (2)	617.5 (.08)^b^	··	··	548.5 (.12)^c^	··	··
Mathematics	8 (3)	7 (2)	7 (1)	480.5 (<.001)^b^	.52	decrease	491 (<.001)^c^	.49	decrease
Reading	8 (2)	8 (2)	7 (2.75)	17 (>.99)^c^	··	··	394.5 (.31)^b^	··	··

^a^Pearson r effect sizes: (a) ·00 indicates no effect size; (b) ± ·.01 to ± ·29 indicates a small effect size; (c) ± ·30 to ± ·49 indicates moderate effect size; (d) ± ·50 to ± 1 indicates large effect size.

^b^Benjamini-Hochberg adjusted significant level due to multiple comparisons is p < 0.025.

^c^Benjamini-Hochberg adjusted significance level due to multiple comparisons is p < 0.05.

#### Assessment of impact from baseline to five months intervention.

The decrease in Avoidance subscale observed at three months assessment of the treatment group disappeared at the five-month assessment, with only a moderate decrease in Functional Impairment remaining. Students at the control school showed a moderate decrease in PTSD scores, along with a decrease in the Avoidance and Arousal subscales. There was no statistically significant difference in scores on the remaining three subscales. Academically, students at the treatment school showed a large increase in both mathematics and reading. Students at the control school showed no change in reading, but a moderate decrease in mathematics. Analysis indicated no significant change in trauma experiences for this time period at either school, removing this as a potentially confounding variable. No adverse events due to participating in the trial were reported.

#### Brief qualitative results summary.

Based on their interviews, the authors of the qualitative study determined that according to the perceptions of 90% of those interviewed, tapping had been helpful in overcoming traumas, improving behavior, and in most cases improving family relationships. They reported that “As the study progressed, several students began to spontaneously apply tapping when they felt anxious, nervous, or worried. They also came to practice it when problems arose in the house, or when they felt impulses of anger or aggressiveness.” According to these authors the principal considered tapping to be a useful tool and recommended the extension of tapping to other schools [21].

## Discussion

Our hypothesis that symptoms of PTSD would be reduced in the treatment group at a greater level than in the control group was not supported. Although a moderate reduction of some trauma symptoms in the treatment group was evident at three months, this was not retained at the five-month assessment, except for decreased scores on the Functional Impairment subscale. The control group showed no changes in trauma symptoms at three months but did show moderate decreases in PTSD and in three subscales at the five-month assessment.

Our second hypothesis was that there would be an improvement in reading and math scores in the treatment group, but a lesser improvement in the control group. This hypothesis was supported with large improvements in both reading and math scores in the treatment group at five months. At five months, the control condition showed no change in reading scores, with a moderate decline in math scores. It is important to note that improved academic performance in the treatment condition corresponded with a decrease in Functional Impairment. Alternatively, students in the control condition showed no change in Functional Impairment, in conjunction with lack of improvement in academic performance. This raises the potential of a link between this subscale and academic performance, which future studies may want to explore. Due to the large improvement in the treatment groups’ reading and math scores, the teachers in the active waitlist control group will be offered training in TFT during the break before the beginning of the 2025 school year.

The authors of the qualitative study commented that group tapping was not sufficient to reduce trauma symptoms in a short period of time. They recommended that tapping be extended over a longer period of time, that better access to individual therapy be available as needed, that parents should be taught to tap, and that the Department of Psychology be made aware of tapping [23].

Limitations included the impossibility of full randomization of students; the attrition rate of students not completing the assessments; and the possibility of other confounding variables between the two schools. For instance, it is possible that a variation in teacher characteristics and competencies between the two groups could explain the differences. A meta-analysis of 40 studies analyzing the effect of teachers on student academic performance found that teacher characteristics and competencies accounted for 9.2 percent of the total variations in academic achievements [31]. It was not possible to measure any differences in culture and differences in community violence between the two schools.

### Conclusion

This feasibility study contributes to the small body of literature on the use of teacher-led classroom interventions in LMICs, where professional resources are scarce and adverse childhood experiences due to personal losses, injuries, interpersonal violence, and community violence are high. Preliminary evidence demonstrated that a brief, daily, teacher-led TFT exercise may increase reading and math skills in adolescents living in conditions of ongoing interpersonal and community violence. However, in these ongoing conditions of violence, a TFT teacher-led group intervention did not maintain a significant reduction of trauma symptoms, and trauma symptoms fluctuated over time.

### Suggestions for further research

Future teacher-led research may need to be conducted over longer periods of time, and include parental education, and ideally, provide additional individual psychological support within the school system [32,33].

## Supporting information

S1 File Appendices available at http://www.blsmanuscript.org.
(DOCX)

S2 File Data Repository available at http://www.blsmanuscript.org.
(PDF)
