# Supplementary material for: A teacher-led classroom intervention in an area of Mexico experiencing community violence: A controlled mixed-method feasibility study
Source: PLoS One. 2025 Jun 9;20(6):e0323562. doi: 10.1371/journal.pone.0323562 (PMC12148136; doi:10.1371/journal.pone.0323562)
Supplement: S1 File — Appendices available at http://www.blsmanuscript.org. (DOCX) [file pone.0323562.s001.docx]

**Supporting Information:**

**S1: Appendix A: Principal’s Form for TFT Group**
https://blsmanuscript.org/english/

(85 KB PDF)

**S2: Appendix B: Principal’s Form for ART-Activity Group**https://blsmanuscript.org/english**/**

(84 KB PDF)

**S3:** [**Appendix C: IRB Signed**](https://blsmanuscript.org/wp-content/uploads/2024/04/IRB-SIGNED-UIIM-DIC22-1.pdf)

https://blsmanuscript.org/english/
(3.4 MB PDF)

**S4:** [**Appendix D: Caretaker’s Consent for TFT group**](https://blsmanuscript.org/wp-content/uploads/2024/04/5-Parent-consent-TFT-Group-Eng.pdf)

https://blsmanuscript.org/english/
(76 KB PDF)

**S5:** [**Appendix E: Caretaker’s Consent for Art-activity**](https://blsmanuscript.org/wp-content/uploads/2024/04/6-Parent-consent-Art-Group-Eng.pdf)

https://blsmanuscript.org/english/
(78 KB PDF)

**S6:** [**Appendix F: Youth Consent form for TFT group**](https://blsmanuscript.org/wp-content/uploads/2024/04/7-Child-consent-script-TFT-Group-Eng.pdf)

https://blsmanuscript.org/english/
(67 KB PDF)

**S7:** [**Appendix G: Youth Consent form for Art group**](https://blsmanuscript.org/wp-content/uploads/2024/04/8-Child-consent-scriptArt-Group-Eng.pdf)

https://blsmanuscript.org/english/
(67 KB PDF)

**S8:** [**Appendix H: Adverse Experience form**](https://blsmanuscript.org/wp-content/uploads/2024/04/9-Adverse-Event-Report-Form.pdf)

https://blsmanuscript.org/english/
(44 KB PDF)

**S9:** [**Appendix I: CATS for Youth**](https://blsmanuscript.org/wp-content/uploads/2024/04/1-CATS-Youth-Eng.pdf)

https://blsmanuscript.org/english/
(343 KB PDF)

**\S10:** [**Appendix J; ATS for Caretakers of Youth**](https://blsmanuscript.org/wp-content/uploads/2024/04/2-CATS-Caretaker-Eng.pdf)

https://blsmanuscript.org/english/
(238 KB PDF)

**S11:** [**Appendix K: Teaching TFT manual**](https://blsmanuscript.org/wp-content/uploads/2024/04/TFT_Algorithm_Manual-for-mexico-traing.pdf)

https://blsmanuscript.org/english/
(698 KB PDF)

**S12:** [**Appendix L: Script for teacher-led intervention**](https://blsmanuscript.org/wp-content/uploads/2024/04/Script-for-teacher-led-intervention.pdf)

https://blsmanuscript.org/english/
(86 KB PDF)

**S13:** [**Appendix M: Assessor Training PowerPoint (download)**](https://blsmanuscript.org/wp-content/uploads/dlm_uploads/2024/04/Mexico-Assessor-training-English.pptx)

https://blsmanuscript.org/english/
(8.9 MB PPTX)
